# Knowledge preservation in the era of big science and AI: strategies for sustainable scientific research

**DOI:** 10.1038/s41467-026-72667-3

**Published:** 2026-05-05

**Authors:** Penn F. Rainford, Annalisa Occhipinti, Bo Wang, Susan Stepney, Claudio Angione, Suraj Verma, Lucia Marucci, Kieren Sharma, Sarah A. Harris, Rudolf M. Füchslin, Edda Klipp, Claire S. Grierson, Mathias S. Weyland, Aalap Mogre, Harold Fellermann, Le Minh Thao Doan, Ioana M. Gherman, Alice L. B. Pyne, Timothy J. Rudge, Victor M. V. Berrelleza, Thomas E. Gorochowski

**Affiliations:** 1 Department of Computer Science, University of York, York, UK; 2 Research IT, IT Services, University of York, York, UK; 3 School of Computing, Engineering and Digital Technologies, Teesside University, Middlesbrough, UK; 4 Laboratory of Pathology, Center for Cancer Research, National Institute of Health, Bethesda, MD USA; 5 School of Engineering Mathematics & Technology, University of Bristol, Bristol, UK; 6 School of Mathematical and Physical Sciences, Sheffield University, Sheffield, UK; 7 ZHAW School of Engineering, Forschungsschwerpunkt Applied Complex Systems Science, Winterthur, Switzerland; 8 European Centre for Living Technology, Venice, Italy; 9 Institute of Biology, Humboldt-Universität zu Berlin, Theoretical Biophysics, Berlin, Germany; 10 School of Biological Sciences, University of Bristol, Bristol, UK; 11 Department of Microbiology, Moyne Institute of Preventive Medicine, Trinity College Dublin, Dublin, Ireland; 12 School of Computing, Newcastle University, Newcastle-upon-Tyne, UK; 13 School of Chemical, Materials and Biological Engineering, Sir Robert Hadfield Building, Sheffield, UK

**Keywords:** Data publication and archiving, Computational biology and bioinformatics, Research data, Research management, Molecular biology

## Abstract

Science is losing knowledge it cannot afford to lose. Negative results go unpublished, hard-won expertise walks out the door with departing researchers, and preservation efforts remain fragmented. The consequences are wasted resources, duplicated effort, and missed discoveries. In this perspective, we argue that the research community can act now by embracing alternative dissemination channels, improving documentation best practices, and building sustainable digital infrastructure. We envision moderated platforms for sharing null results and practical know-how, community-driven standards, and AI-powered tools that lower barriers to implementation. With coordinated effort, science can become more open, efficient, and resilient for future generations.

## Abstract (web summary)

Science routinely discards valuable knowledge, from unpublished negative results to the tacit expertise lost when researchers move on. Here, the authors propose a roadmap for preserving this hidden knowledge through community-driven platforms, open standards, and AI-assisted documentation.

## Introduction

Modern scientific research is undergoing an unprecedented transformation in the scale and nature of the data it produces, challenging traditional approaches to knowledge preservation and dissemination. This shift is driven by several converging forces that have reshaped research practice over the past decade. In the biosciences, our area of expertise, laboratory automation has expanded dramatically^1^, enabling experiments at scales previously unimaginable, while innovations in high-throughput methods have revolutionized the depth and breadth of data that can be collected^2–4^. Simultaneously, advances in computational power have enabled the development of increasingly sophisticated mathematical models, leading to large-scale simulations that generate vast quantities of theoretical predictions^5–8^ or design entirely new biological components and systems from scratch^5,9,10^.

This growth in data has been accompanied by a notable shift towards interdisciplinary approaches, where breakthroughs increasingly draw on experimental, computational, and analytical methods from multiple scientific fields to open new lines of inquiry on long-standing questions^5^. The data being produced is therefore not merely greater in quantity but fundamentally different in character. Modern research generates heterogeneous, multi-modal datasets that combine wet laboratory experiments with computational simulations and extend beyond traditional disciplinary boundaries^11^. Such datasets often require specialised software, custom analysis pipelines, and domain-specific expertise to be interpreted effectively. The complexity and interdependencies within these data also create new challenges for long-term storage, sharing, and preservation^12^. These difficulties are mirrored in the experimental sciences, where laboratory freezers are filling with genetic constructs, cell lines, and other biological materials that represent invaluable research assets but are increasingly difficult to catalog, maintain, and share^13^.

Peer-reviewed publication remains the traditional model for capturing and disseminating scientific knowledge, but this approach is increasingly recognized as inadequate^14^. Conventional journals impose strict page limits, leading to abbreviated descriptions of methodologies that omit crucial contextual information and troubleshooting insights needed for true reproducibility. Digital supplements and links to external repositories can partially address these shortcomings, but they remain secondary when they should now be standard. These additional elements are often incomplete, poorly maintained, and difficult to locate. These challenges are further compounded by high researcher turnover, a longstanding issue that has become more pronounced in recent years due to competitive job markets, funding constraints, and increasing career mobility^15^. The loss of knowledge and expertise as people move between roles impairs research efficiency and significantly hampers scientific progress.

Given this shifting landscape, we are rapidly approaching a point where new methods for knowledge preservation and dissemination become essential. The inability of current systems to effectively capture, organize, and share the full breadth of modern research outputs threatens to undermine the cumulative nature of scientific progress. In this perspective, we argue that the time is ripe to pursue more innovative approaches to sustainable knowledge preservation, with a focus on applications in the biosciences. After considering the core issues, we discuss how to effectively grow and integrate community-driven platforms and open science initiatives that offer more inclusive paths to knowledge sharing. Such developments would position us to build a more robust, resilient, and equitable system for preserving scientific knowledge, capable of meeting the growing demands of modern research while maintaining the rigorous standards for scientific integrity.

## Key Challenges

The reproducibility and reusability crisis in modern scientific research stems from fundamental inadequacies in how research outputs are documented, preserved, and shared^16,17^. Current publication practices often fail to provide sufficient detail for true reproducibility, omitting procedural nuances, full parameter settings, and the practical insights that determine experimental success. This documentation gap is compounded by inadequate storage and publication of related elements, such as data, programming code, detailed method documentation and metadata (i.e., data describing features of other data), for physical items such as strains. These are essential for reproducibility but exist outside traditional publication frameworks. This issue is well recognized, and many well-designed methods for disseminating different outputs suffer from their disparate locations and inconsistent metadata, making them difficult to locate, access, and use effectively. Long-term preservation faces further challenges from failing freezers leading to denaturation and degradation of samples, evolving file formats, deprecated software dependencies, and the absence of sustained funding for maintenance^18^.

These issues are further compounded in interdisciplinary research due to misunderstanding and confusion around core terminology, with common terms such as ‘in vivo’, ‘in vitro’, and ‘gene’ having differing meanings across research communities. A more complete and shared common lexicon could potentially help, but poses its own challenges in terms of adoption and the need for many terms to only make sense in a broader context that is difficult to formalize.

The reusability of research outputs presents even greater difficulties, as the utility of shared resources often degrades over time due to inadequate curation and maintenance^19^. Biological materials stored in laboratory freezers become mislabelled, contaminated, or lost entirely as record-keeping systems fail. Code that was functional at the time of publication becomes obsolete as underlying computational environments change, and the accompanying documentation is frequently insufficient for others to use without direct communication with the original authors. This problem only worsens as time passes and researchers move on.

Null results (i.e., findings that do not support an experimental hypothesis or fail to replicate existing work) are systematically excluded from the published literature despite their recognised scientific value^20^. Bias towards significant, novel, and successful outcomes among editors, reviewers, and authors^21,22^ creates a distorted view of research that misleads meta-analyses, wastes resources through repeated failed attempts, and hinders the development of more accurate theoretical frameworks. The wealth of additional data generated by computational models, including calibration results, parameter sensitivity analyses, alternative model formulations, and extensive simulation outputs, rarely finds its way into publications. Similarly, supplementary experimental data, secondary measurements, and exploratory analyses that provide valuable context for interpreting primary results are often relegated to brief mentions or omitted entirely. While these data may be less rigorously validated (e.g., null results may lack sufficient controls), they may still help to guide future experiments as long as their limitations are well understood. For this reason, these types of results should still be disseminated through appropriate channels and linked to published research to ensure they can inform future meta-analyses and reviews.

The loss of skills and tacit knowledge through personnel turnover represents one of the most significant yet underappreciated challenges facing scientific research^23^. When experienced researchers depart, they take with them subtle technical insights, methodological refinements, and practical knowledge that are often essential to the successful execution of complex procedures. Equally valuable is knowledge of what has been attempted but did not work, including initial investigations that led nowhere, experimental approaches abandoned due to technical difficulties, and theoretical frameworks that proved unproductive. This information about failed attempts could save future researchers considerable time and effort but typically exists only in the memories of individuals. Such knowledge is also poorly suited to dissemination through traditional publication channels, as it has not been validated or checked for errors in the same manner as published findings.

The computational aspects of modern research are particularly vulnerable to the loss of tacit knowledge. Understanding why certain parameter combinations produce unstable results, how to interpret ambiguous model outputs, or which computational approaches are best suited to specific types of data represents specialized knowledge that is difficult to codify but essential for effective research. The software engineering skills required to keep computational systems functional represent an additional vulnerability, as the departure of technically skilled individuals can leave research groups unable to maintain or modify essential tools. As computational methods grow increasingly complex, this technical knowledge becomes ever more specialized and difficult to replace, creating dependencies that can severely disrupt research continuity when key personnel leave.

The challenges discussed so far are closely connected to the well-recognized issues of open data and open research, and they share the same fundamental constraint: researcher time is limited. Most researchers cannot devote substantial time to open science initiatives whose effectiveness remains unproven^24,25^. This problem is compounded by the tendency to treat each challenge and proposed solution in isolation rather than as part of a broader issue that the community as a whole must address. If additional work could be integrated into existing workflows and minimized through system and approach compatibility, the effort required would be reduced, paving the way for tools that further simplify the process. Achieving this requires acknowledging that a generation of researchers will need to invest their time in these new efforts, and that the intrinsic value of establishing their effectiveness must be recognized before institutions can be expected to formally reward them.

As outlined above, the preservation and dissemination of knowledge in modern research requires coordinated action on multiple fronts. Rather than waiting for comprehensive system overhauls, the scientific community can begin implementing targeted interventions that build on existing infrastructure while establishing the foundations for more sustainable practices. These immediate actions focus on harnessing current capabilities, reshaping cultural norms, and developing new standards that can evolve as technologies advance.

## Integrating existing solutions

Open science initiatives have prompted a wave of community-driven efforts to improve knowledge preservation and reproducibility (Tables 1 and 2). Although these existing solutions only partially address the challenges and are unevenly adopted, they represent tangible steps towards strengthening scientific continuity and offer a glimpse of what a more integrated ecosystem might look like.

**Table 1:** Common repositories for physical and digital research items in the biosciences

Name^a^	Items stored	Ref.	URL
**Physical biological resources**			
Addgene	Plasmids and strains.	^41^	www.addgene.org
ATCC	Microorganisms and cell lines.	^69^	www.atcc.org
BioBricks Registry	Biological parts and characterisation data.	—	registry.igem.org
DSMZ	Microorganisms and cell lines.	—	www.dsmz.de
SEVA	Standardised plasmids (SEVA format).	^70^	seva-plasmids.com
Svalbard Seed Vault	Global long-term seed storage facility.	^71^	www.seedvault.no
UK Biobank	Large-scale biomedical samples resource.	^72^	www.ukbiobank.ac.uk
**Experimental data resources**			
ArrayExpress	Functional genomics data.	^73^	www.ebi.ac.uk/arrayexpress
BioSamples	Metadata about biological samples.	^74^	www.ebi.ac.uk/biosamples
DDBJ	DNA and RNA sequence data.	^75^	www.ddbj.nig.ac.jp
EcoCyc	Genomic, metabolic, regulatory data.	^76^	www.ecocyc.org
EMDB	Electron microscopy data	^77^	www.ebi.ac.uk/emdb
ENA	Nucleic acid sequences.	^78^	www.ebi.ac.uk/ena
Gene Ontology	Knowledgebase for the functions of genes.	^37^	geneontology.org
GTEx	Tissue/cell-specific gene expression & regulation.	^79^	gtexportal.org
KEGG	Genomic, metabolic, and regulatory data.	^80^	www.genome.jp/kegg
MMDB	Molecular dynamics simulations.	^27^	mddbr.eu
NCBI	Wide variety of sequence and expression data including the hosting of other key repositories like GenBank, SRA and GEO.	^54^	www.ncbi.nlm.nih.gov
PDB	Protein structures.	^81^	www.rcsb.org
PDB-IHM	Integrative molecular structures	^82^	pdb-ihm.org
RegulonDB	Transcriptional regulation data for *E. coli* K-12.	^83^	regulondb.ccg.unam.mx
SynBioHub	Genetic parts and designs for synthetic biology.	^32^	synbiohub.org
UniProt	Protein sequences and annotations.	^84^	www.uniprot.org
**Models, workflows and code**			
AiiDA	Automated interactive infrastructure and database for computational workflows.	^43^	www.aiida.net
BioModels	Models of diverse biological systems.	^85^	ebi.ac.uk/biomodels
Galaxy	Bioinformatics workflows.	^40^	galaxyproject.org
GitHub	Computer code and documentation.	—	www.github.com
**Protocols, pre-prints, and other research resources**			
bioRxiv	Pre-print server for the biological sciences.	^86^	www.biorxiv.org
Dryad	General research data repository.	^87^	datadryad.org
Figshare	Repository for datasets, figures, and other outputs.	^88^	figshare.com
JoVE	Videos of experimental protocols.	—	www.jove.com
Protocols.io	Step-by-step experimental protocols.	^63^	protocols.io
Zenodo	Open-science repository.	^89^	zenodo.org

^a^ Acronyms for repositories given where commonly used. *ATCC* American Type Culture Collection, DSMZ: German Collection of Microorganisms and Cell Cultures, *EMDB* Electron Microscopy Data Bank, *ENA* European Nucleotide Archive, *GEO* Gene Expression Omnibus, *GTEx* Genotype-Tissue Expression, *KEGG* Kyoto Encyclopedia of Genes and Genomes, *JoVE* Journal of Visualised Experiments, *MDDB* Molecular Dynamics Data Bank, *NCBI* National Center for Biotechnology Information, *PDB* Protein Data Bank, *PDB-IHM* Protein Data Bank for Integrative and Hybrid Methods, *SEVA* Standard European Vector Architecture, *SRA* Sequence Read Archive.

**Table 2:** Communities supporting sustainable research practices in the biosciences

Name	Description	Ref.	URL
Benchling	Cloud-based platform that combines data management, protocol sharing, and collaboration tools to improve reproducibility and enable more efficient lab workflows.	^90^	benchling.com
BioStars	Bioinformatics and computational biology forum facilitating knowledge exchange, troubleshooting, and collaborative problem solving.	^64^	biostars.org
COMBINE	Standards for reproducible modelling across all areas of computational biology.	^91^	co.mbine.org
Cultivarium Portal	AI-enhanced portal to support creation of collaborative networks that share data, discussions, and new findings (including negative results) for diverse organisms.	—	portal.cultivarium.org
Data Hazards	Open community aiming to identify the hazards of using data science for interdisciplinary research	^92^	datahazards.com
FAIRsharing	Standards, repositories, and best practices for data management plans, publishing, or collaboration.	^26^	fairsharing.org
FORCE11	Community of scholars, librarians, archivists, publishers and research funders that help facilitate improved knowledge creation and sharing.	^93^	force11.org
GA4GH	Not-for-profit global alliance for genomics and health aiming to set standards and policies to expand genomic data use within a human rights framework.	^94^	www.ga4gh.org
GRN	Cross-disciplinary consortium that aims to increase trustworthiness and transparency of scientific research.	—	reproducibilitynetwork.de
ResearchGate	Social network for scientists to share publications, answer questions, and track impact metrics.	^65^	www.researchgate.net
SBOL	Community aiming to build standards, tools and tutorials for simplifying the representation and visualisation of genetic design information.	^34,35^	sbolstandard.org

^a^ Acronyms for communities given where commonly used. *COMBINE* computational modelling in biology network, *GA4GH* Global Alliance for Genomics and Health, *GRN* German Reproducibility Network, *SBOL* Synthetic Biology Open Language.

Several frameworks have emerged to guide best practices in data and model stewardship. The FAIR principles aim to ensure that data are findable, accessible, interoperable, and reusable, and have become a widely accepted benchmark for evaluating how research outputs should be structured and shared^26,27^. BioFAIR adapts this approach for the life sciences by incorporating metadata standards, provenance tracking, and reproducibility layers. For computational models, the recently proposed CURE principles^28^, which focus on the need for models that are Correct, Unbiased, Robust, and Explainable, highlight the importance of transparency and validation in ensuring long-term trust and reuse. Together, these frameworks offer not just guidance but practical benchmarks for assessing the current ecosystem of repositories, tools, and platforms.

Specialised data repositories are commonplace in the biosciences. Examples include the Protein Data Bank (PDB)^29^, GenBank^30^, the European Nucleotide Archive (ENA)^31^, and SynBioHub^32^, all of which support data preservation within specific domains through internationally coordinated, community-driven curation and validation. Alongside these repositories, standardisation efforts have produced file formats such as FASTA, the General Feature Format (GFF)^33^, the Synthetic Biology Open Language (SBOL)^34,35^ and macromolecular crystallographic information file (mmCIF)^36^, as well as structured annotation systems like the Gene Ontology (GO)^37^. These standards have been central to enabling automation, reuse, and improved interoperability^38^, demonstrating the effectiveness of community-developed standards and their adoption. However, efforts to integrate these into traditional publications have so far treated them as exceptions rather than the norm.

Version control systems, particularly Git and platforms such as GitHub and GitLab, have transformed computational reproducibility by enabling code tracking, collaboration, and transparent records of software development^39^. These tools can document final versions, release cycles, and development processes, and when combined with issue tracking and automated testing frameworks, they create rich environments for computational research. However, adoption remains uneven, and many researchers lack the training to use these tools effectively. Moreover, version control systems rarely connect directly to experimental protocols, metadata, or semantic standards, limiting interoperability and automated reuse. The commercial nature of several major repositories also raises questions about the longevity of these resources and the factors governing their future development and access. In the area of analysis code, bioinformatics workflow platforms such as Galaxy^40^ enable reproducible pipelines through containerized, GUI-based environments, particularly for standardized tasks in genomics. However, many analyses remain highly bespoke, relying on custom code, ad hoc toolchains, and dataset-specific parameters that resist integration into such structured systems.

In the experimental domain, the non-profit organization Addgene supports biological resource sharing through a centralized platform for distributing plasmids and other molecular tools, complete with documentation, quality control, and user feedback^41^. Its success stems from strong curation standards, usability, and a sustainable funding model in which users pay a reasonable fee for the service. Systems like LabArchives enable metadata-rich documentation of experimental protocols, including detailed records of failed procedures along with analysis of potential causes and lessons learned^42^. In the computational sciences, systems such as AiiDA have been developed to better track and manage the complexity of large-scale automated computational workflows^43^. Complementing these efforts, AI-based summarisation tools can systematically index technical missteps and surface potentially error-prone approaches, creating searchable databases of troubleshooting knowledge to guide future research. Platforms such as CellRepo^44^ and OpenBioSim^45^ are also beginning to embed experimental lineage and quality flags that preserve information about deprecated cell lines or unstable simulations, extending access to these resources even when they are no longer actively maintained^44^.

While these existing solutions are effective at addressing individual issues, they often function in isolation and are characterized by inconsistent interfaces, incompatible standards, and insufficient attention to long-term sustainability. As a consequence, there is a pressing need for infrastructure that enables structured reuse, scalable moderation, and interpretability—both by researchers and by the emerging systems designed to support them, such as AI assistants. The first and most fundamental shortcoming is findability. Most researchers rely on published papers to discover work aligned with their interests, yet links to associated outputs are often buried in footnotes or mentioned inconsistently. At a time when digital deliverables are an expected component of research, it should be standard practice to include a dedicated section, on equal footing with a bibliography, for linking to related resources. This section should not contribute to paper length and should be standardized in a manner similar to a reference list.

Such a development would also begin to address the second major issue: maintenance. This challenge exists at many levels, from the individual outputs attached to a project to the storage platforms themselves. Including links to all outputs of a publication would allow links to be checked, flagged when broken, and updated by authors. Combined with existing efforts to improve standards for experimental data and code, this would enable broader use of automated maintenance, including AI-driven tools that keep software up to date with advances in programming languages and supporting libraries. In this way, adoption of standards and existing systems will help maintain the value of past research and its outputs by giving them longevity and ongoing relevance. Making these resources more findable and accessible will also increase their chances of wider adoption, making them easier to sustain under current funding models.

The maintenance and adoption of existing systems ultimately depend on increased funding and institutional endorsement, which are likely to follow from their integration into research workflows and funding models. Universities and funders can incentivize the use of these platforms by recognizing contributions in funding and promotion decisions. Professional societies can promote adoption through case studies, training, and community standards. Addressing practical adoption barriers, including poor usability, limited integration, and unclear contribution guidelines, would further accelerate uptake.

## New platforms

### Results of all types

The exponential growth of research data, combined with the fragility of conventional knowledge dissemination systems and the persistent loss of contextual information at the interface between experiments and computation, has created an urgent need to address a critical dimension of research sustainability: the systematic preservation of scientific failure. Scientific progress depends as much on understanding what does not work as on celebrating what does, yet current academic publishing infrastructure systematically penalizes, obscures, or discards failed experiments, unstable computational models, and null results. This is especially problematic in the sciences, where the iterative nature of research means that failed experiments often contain crucial insights for future efforts^46^.

This problem is fundamentally rooted in how the research community defines and rewards “results” within the current publication paradigm. Academic publishing overwhelmingly favors significance and novelty, and to some extent, confirmatory findings, creating powerful incentives for researchers to focus their dissemination efforts on positive outcomes^47^. This systematic bias produces a distorted record of scientific activity that is both misleading and wasteful, as researchers unknowingly repeat failed efforts that were never shared with the broader community.

The consequences of this selective preservation extend far beyond individual research projects. Meta-analyses become skewed towards positive findings, creating inflated effect sizes and a misleading sense of research consensus. Machine learning models trained on this biased literature inherit and potentially amplify these distortions^48^. Perhaps most critically, researchers repeatedly undertake procedures that have already been shown to fail, wasting time, resources, and intellectual effort that could be directed towards more promising avenues if knowledge of previous failures were accessible.

Currently, only a few journals, such as PLOS ONE and the Journal of Negative Results in BioMedicine, encourage the publication of null or negative results within the standard peer review system^49^. Moderated but non-peer-reviewed platforms could offer a more open environment for researchers to share null results that would be unsuitable for a journal context, such as minor observations. Moderation on these platforms should focus on methodological soundness rather than novelty or significance, allowing valuable negative findings to contribute to the cumulative scientific record. Dedicated platforms for null results and brief reports on failed experiments, including methodologies and lessons learned, could assign permanent digital object identifiers (DOIs) to ensure the work can be cited, while also formally recognizing the value of failed experiments. The effort to establish and maintain such a platform would depend on context. However, for a moderate-sized system that makes use of existing cloud-based infrastructure (e.g., AWS), it is likely to require several years of a post-doctoral researcher’s time, in addition to some input from a senior academic during development, and then a fraction of a post-doctoral researcher’s time (e.g., 30%) once up and running to maintain the resource. Such costs would be within the realm of most learned societies, with them also best placed to establish the supporting communities around a resource as well as any underpinning standards and wider culture.

### Distributed storage

Rather than pursuing the development of a single monolithic repository, which would face inevitable challenges of scale, governance, and sustainability, the research community should embrace a federated, community-maintained approach^50^. This vision encompasses a distributed library spanning research domains, individual laboratories, and existing platforms, with curation responsibilities shared between human experts and intelligent computational agents. Efforts towards distributed data sharing are not new; the now-defunct BioTorrents^51^ attempted to create such a platform over a decade ago. Torrent-based sharing is a proven approach for distributed storage, with built-in curation: only data deemed relevant and actively maintained is reseeded. While that particular initiative did not succeed, the underlying principle remains sound: distributed storage and shared maintenance responsibilities have the potential to reduce overheads and increase the resilience of storage infrastructure, particularly in the current era of cloud computing.

### Interconnected and searchable systems

Combining these types of storage with existing preprint servers^52^, micro-publications^53^, institutional repositories, community wikis, and domain-specific databases^54^ would allow for the dissemination of null results, small replication studies, and methodologies that are not suitable for inclusion in typical peer-reviewed publications. Embracing this positive view of failure would represent a fundamental shift in how the scientific community conceptualizes knowledge preservation, moving from a model that celebrates only successes to one that recognizes the essential value of comprehensively documenting all findings. By ensuring that no experiment is wasted, regardless of how inconclusive or disappointing, we create a more complete, honest, and sustainable record of scientific activity, while also providing practical resources that can accelerate future research and better support the complex, interdisciplinary challenges facing contemporary science. Funders, tenure committees, and professional societies must formally validate these channels as legitimate scholarly contributions and reward researchers accordingly. Publishers also have a critical role to play and should require authors to release complete datasets, analytical code, and supporting documentation as part of the publication process. This practice not only enhances research integrity but also facilitates the preservation of knowledge that may prove valuable in ways not initially anticipated. While raw data may be short-lived, metadata, code, and lessons from failed experiments often hold long-term value. A tiered preservation model (e.g., short-term for raw data, medium-term for processed outputs, and long-term for reusable data and methods) could balance preservation needs with cost and complexity, particularly given the extensive storage requirements of large datasets. Expert curation will be key to making these value-based decisions.

Metadata repositories could serve as a connective layer, linking past and future research outputs to one another and to the relevant publications and documentation. The ability to associate all null and minor results generated alongside a published article with a single set of analyses and data would significantly support open science initiatives while reducing time overhead by providing a central resource for creating entries, managing metadata tags, and maintaining resources. Other platforms, such as fan fiction archives^55^, have demonstrated that moderated tag-based systems can sustain a common terminology within complex and dynamic fields. Moderation does introduce an additional time requirement. However, this burden can be reduced by incorporating tagging into the review process and delegating moderation of tags to journal editors and program committees within the relevant fields, who already consider keywords and subjects for conference programs and special editions. This decentralized approach would enable community-driven consensus on terminology, improving accessibility and the searchability of research. In the future, these platforms could seamlessly connect code repositories, experimental protocols, biological materials, and analytical datasets, creating comprehensive research packages that enable true reproducibility and serve as an extension of published work.

## Individual knowledge preservation

Personal insights gained during scientific research represent another valuable yet vulnerable form of knowledge. Modern research demands sophisticated technical skills across multiple domains, from navigating complex protocols and mastering specialized software to developing custom computational pipelines and integrating diverse data sources using often undocumented methods. This context-dependent expertise requires an understanding not only of standard procedures but also of when and how to adapt them to specific research questions. Such knowledge is particularly fragile during researcher transitions between projects, institutions, or careers, making an understanding of how it is created essential for developing effective preservation strategies. Dedicated time for documentation and knowledge transfer at these transition points is vital.

Loss of this knowledge is common in rapidly evolving fields where techniques change faster than documentation can keep pace. The transition from academia to industry represents a significant drain, as researchers may be unable to share proprietary techniques or may simply lose contact with academic networks. The prevalence of short-term contracts and temporary academic positions creates frequent disruptions to knowledge transfer and prevents the development of long-term mentoring relationships that foster best practice^56,57^. The pressure to publish or perish is particularly acute for those constantly focused on securing their next position, often resulting in sudden departures with little or no handover or documentation. Where possible, it may be better practice for institutions to make greater use of permanent technicians rather than rely on short-term contracts, helping to increase the pool of permanently employed personnel at research institutes and reduce reliance on short-term researcher contracts.

Sharing personal insights also requires platforms capable of capturing both explicit knowledge and tacit expertise. These platforms should support a range of content types, including videos, annotated protocols, and interactive tutorials, which can convey complex procedures more effectively than text alone. The platform architecture should facilitate community validation and iterative improvement of shared content, creating living resources that evolve with a community’s collective experience. Community support mechanisms are needed to allow members to flag content for review while maintaining access to shared resources. Such platforms should also incorporate version control to track the progression of shared knowledge and preserve access to historical versions. Progress is being made in this space; for example, the PRISM system developed by Cultivarium allows researchers to discuss and demonstrate a protocol while wearing a headset that records the process on video. PRISM then uses AI to generate an annotated video and text-based protocol with minimal effort, better capturing the nuances involved in each step.

All of these methods for improving knowledge preservation require time, much like developing new platforms, running conferences, and participating in the research community. Researchers prioritize their time based on incentives and policy. However, certain activities are undertaken as part of academic citizenship, such as peer review, and time for these is permitted because they are expected of academics. The UK’s Researcher Development Concordat, which has broad support from institutions and funders, provides 10 days per year for professional development. Despite its clear benefits for career progression, it has often been overlooked by researchers themselves.

Establishing dedicated time for knowledge preservation activities could be an immediate and practical step towards addressing our key challenges. This time could be spent on community activities, documenting work, and maintaining data and methods from previous projects. Requiring researchers to account for a specific amount of knowledge preservation activity would also enable institutions and funders to consider these contributions in promotion and grant decisions. The benefits of open science and improved long-term stewardship of knowledge and data should be clear to institutions and funders alike, and simple incentives such as protected time can be highly effective in driving these practices forward.

More broadly, a shift in the mindset of all researchers is required. Producing high-quality methodologies, protocols and results takes significant time and effort. However, such resources are far more valuable to the community, and their use long outlives the initial outlay in effort. Learned societies, universities and funders have a role to play in demonstrating this and reshaping priorities and culture across fields from purely valuing the amount of research output, to instead fostering fewer outputs of higher quality that include good documentation, detailed controls/validation and distribution through channels that will support long-term access and use.

Education is one of the most actionable ways to improve knowledge preservation. Targeted programs for both early-career and established researchers can address critical skill gaps. Workshops and summer schools should combine theory with hands-on training in documentation and data management, with reproducibility placed at their core. Online tools, tutorials and videos, such as those hosted by the Journal of Visualized Experiments (JoVE) and IRIS^58^ (a subject-specific tool repository), can broaden access and support just-in-time learning for specific protocols, tools, and standards. Collaborative activities such as coding groups, hackathons, and peer mentoring can help embed best practices while fostering community connections that support long-term adoption. Workshops and summer schools can also provide the time and space for researchers to contribute to these online resources themselves, creating a virtuous cycle of shared knowledge.

Peer-mentoring programs can provide ongoing support for researchers adopting new knowledge preservation practices, offering both technical guidance and encouragement through what can be challenging transitions. Mentoring should be bidirectional, recognizing that all participants bring valuable knowledge while also having areas where they can benefit from guidance. AI-powered systems can further enhance these programs by identifying optimal mentor-mentee pairings based on research interests, technical skills, career stages, and learning objectives^59^.

A key component of this educational effort is cross-disciplinary training. As research becomes increasingly data-driven, bioscientists must develop foundational skills in areas such as AI and software development, while computational scientists need greater fluency in domain-specific research contexts. Bridging these gaps will foster more effective collaboration and improve the design and usability of preservation systems across disciplines. Education must also address institutional knowledge loss through clear expectations around researcher departures, comprehensive lab records, and the systematic capture of lessons learned. Beyond allocating time, dedicated spaces and formats for creating this documentation are equally important; writing retreats, doc-a-thons, and workshops could all be run regularly to support researchers in this work.

## Community knowledge and learning

The transfer of hands-on technical knowledge relies heavily on community connections and networks, and the scientific community has already developed effective models for fostering them. Scientific societies play a key role in nurturing these networks by organizing research conferences, workshops, and summer schools that give researchers opportunities to learn new techniques, share experiences, and build lasting professional relationships. The informal networking at these events is often as valuable as the formal presentations, helping researchers identify collaborators, mentors, and peers who can support their ongoing development. Such networks are relatively inexpensive to maintain but depend on goodwill within the research community and continuity through stable funding.

Establishing sustainable standards for knowledge preservation requires extensive community engagement through structured workshops, collaborative publications, and iterative refinement. Effective standards cannot be imposed from above; they must emerge from the collective wisdom and practical experience of the research community. To support this process, workshops should prioritize inclusive participation, actively targeting new attendees to ensure fresh perspectives and prevent the formation of insular expert groups. Workshop design should incorporate multiple formats to address different aspects of standards development. Technical sessions should focus on specific tools, platforms, or methodological approaches, while strategic sessions should address broader questions of incentives, governance, and community adoption. Cross-disciplinary collaboration should be actively encouraged, recognizing that scientists from different disciplines approach problems in fundamentally different ways. For example, biologists are typically hypothesis-driven, focusing on experimental design, phenotypic characterization, and mechanistic interpretation, while computational scientists often prioritize algorithm development, data integration, and model generalization. Cross-disciplinary workshops and projects should adopt co-design principles in which experimentalists and computational researchers jointly define objectives, ensuring that the data generated is suitable for computational analysis and that computational models address biologically meaningful questions. Such workshops can identify common challenges and opportunities for standardisation across research domains, while discipline-specific sessions can address the unique requirements of particular fields^60^.

Synthesizing workshop discussions into broader community guidelines requires systematic documentation and analysis of emerging themes, disagreements, and points of consensus. This process should involve both the immediate capture of workshop outputs and subsequent analysis to identify patterns across multiple relevant events and communities. Feedback from researchers who could not attend should also be incorporated, ensuring that standards development remains inclusive and representative. Signposting tutorials represent a particularly valuable workshop output, providing researchers with awareness of available resources without requiring comprehensive training in specific tools or methods. These tutorials should help researchers identify appropriate resources for their needs while providing sufficient information to evaluate the relevance and quality of different options. An excellent example of this community-driven approach is COMBINE (COmputational Modelling in BIology NEtwork), which coordinates the development of various community standards and formats for computational biology (Table 2).

While in-person community-building is essential, several factors limit its effectiveness in transferring technical knowledge equitably and sustainably. Conference attendance is often restricted by financial constraints, geographic limitations, and competing priorities, creating barriers that disproportionately affect early-career researchers and those from resource-limited institutions. The emphasis on presenting positive results may also discourage the sharing of negative findings or failed experiments, despite the valuable learning opportunities these provide. Workshops and summer schools frequently have limited capacity, and selection processes can inadvertently create additional barriers to participation. The intensive nature of these programmes, while conducive to deep learning, can be inaccessible to researchers with teaching responsibilities, caring obligations, or other commitments that make extended travel difficult. These events also rely heavily on community experts volunteering their time, which can be burdensome alongside other responsibilities and often goes unrecognised beyond the immediate beneficiaries.

To address these limitations, the scientific community has developed a range of platforms for sharing technical expertise, several of which now represent mature and well-established resources. Our aim is not to propose replacements for these efforts but to advocate for their wider adoption and integration into a more coherent framework. Code carpentry initiatives, such as Software Carpentry and Data Carpentry^61,62^, offer practical, hands-on training that teaches computational skills and best practices to researchers across disciplines, bridging the gap between theoretical understanding and implementation. Wiki-based platforms have proved particularly effective for the collaborative documentation of protocols, troubleshooting guides, and methodological insights (e.g., see Cultivarium Portal, Table 2). Unlike static documents, wikis support iterative improvement, enabling communities to capture evolving best practices and emerging techniques. Platforms such as protocols.io^63^ have demonstrated the value of structured, version-controlled documentation of experimental procedures that can be shared, modified, and refined by the broader community. Stack Overflow-style Q&A platforms have also become indispensable for the computational aspects of research, providing searchable databases of solutions to common problems. Specialised platforms such as Biostars^64^ for bioinformatics and ResearchGate^65^ for broader scientific questions have adapted this model to domain-specific needs, creating communities where researchers can seek and share solutions to technical challenges.

Community-developed guidelines represent a crucial milestone in standards development, but their format and governance require careful consideration to ensure long-term relevance. Traditional publications are poorly suited to capturing information that must evolve in response to technological advances and changing community needs. Living documentation offers a more effective alternative: version-controlled resources that can be updated systematically as new technologies emerge, methodological approaches develop, and community consensus shifts. Version control is essential not only for keeping guidelines current but also for preserving access to historical versions, allowing researchers to understand how practices have evolved and to follow guidance appropriate to their specific context. Governance structures should be established to resolve conflicts between research communities or disciplinary approaches and to maintain the coherence of shared standards. Supporting materials, including practical tutorials, implementation guides, and troubleshooting resources, should be integrated with core guidelines while maintaining their own update cycles, enabling rapid responses to emerging technical issues or user feedback.

## Building value

The success of knowledge preservation initiatives depends on participation by both individuals and the community as a whole. To build value and sufficiently incentivize researchers to invest time in these activities, we need to demonstrate their effectiveness, champion them within the community, and advocate for recognition and reward from funders and institutions. The goal is to establish sustainable incentive structures that reward participation while reducing the effort required. This approach recognizes that cultural change typically begins at the community level but requires both positive reinforcement for desired behaviors and the removal of barriers that prevent adoption.

The development of robust standards and comprehensive training programs can guide best practices, helping reduce the likelihood that individual researchers reinvent the wheel. However, these systems must accommodate differences in methodological approaches, providing flexibility within a structured framework. For this reason, we do not propose a ready-made solution to the key challenges outlined in this work. No solution can succeed without community endorsement, and this is far more likely when researchers are engaged early in the development process. Many promising open science initiatives have faltered because they were developed by a small group unable to account for the broader needs of the wider community.

Short-term incentives are essential to achieving the critical mass needed to drive long-term cultural change. These might build on existing mechanisms such as community recognition programmes for exemplary knowledge sharing, funding opportunities for researchers who demonstrate commitment to open practices, and professional development tied to community engagement. Such incentives should be expanded and promoted at all career levels and stages. The incentive structure should also leverage the growing momentum behind open data and open science, positioning comprehensive knowledge preservation as a natural extension of current best practices. Crucially, recognition and reward for these activities must come early and enthusiastically from institutions, sustaining rather than dampening momentum so that new systems have the best chance of success.

AI offers significant potential for reducing effort by automating routine tasks, generating initial drafts of documentation, and providing rapid quality assessments of shared resources. However, the deployment of these technologies must be carefully managed to ensure accuracy, prevent bias, and maintain human oversight of critical decisions. AI systems should be designed to augment human capabilities rather than replace human judgment, providing tools that make comprehensive documentation more feasible without entirely removing human responsibility for quality and accuracy. This principle is long-standing; an IBM training manual from 1979 warned that “a computer can never be held accountable, therefore a computer must never make a management decision.” Important decisions should always retain human oversight.

The challenge of industry partnerships and commercially focused research requires careful consideration in the design of incentives. While concerns around intellectual property and competitive advantage are legitimate, incentive structures should encourage researchers to share non-proprietary knowledge while respecting genuine confidentiality requirements. Tiered sharing systems could address this by allowing researchers to share methodological insights and negative results while protecting commercially sensitive information.

A central aim should be to cultivate intrinsic community value, as in open-source software development, where individual contributions generate collective benefits that exceed the sum of each person’s effort. Achieving this requires building cultural norms that recognize knowledge sharing as both a professional responsibility and a source of scientific prestige. The open-source model offers valuable lessons in community governance, quality control, and incentive design that can be adapted for scientific knowledge preservation, including transparent contribution processes, merit-based recognition systems, and collaborative decision-making structures that ensure community ownership of shared resources.

Building an effective and efficient incentive design should profit from what is known from behavioural economics and management science. Mitigating the tension between individual interests and value creation on the community level is far from trivial and requires a somewhat sober perspective on the driving forces behind human activities (e.g., for risk taking see ref. ^66^). Moreover, there exist models of tacit knowledge in organizations that capture the fact that knowledge is not only stored in individuals, but also in the way in which they are connected (e.g., transactive memory systems in the context of research groups^67^). There is, however, a subtle danger: most studies in management science seem to focus on technological innovation and engineering. Knowledge preservation in engineering deals with human-designed structures that are generically modular and organized using well-defined interfaces. Description and segmentation of biological knowledge, which represents evolved structures, may well be more demanding. Best practices adequate for systems that can be fully represented by blueprints and spec sheets are not necessarily optimal for biological information.

Developing community value also requires engaging with the broader institutional and policy context that shapes research practices. This means advocating for changes to promotion and tenure criteria, funding agency requirements, and professional society standards so that comprehensive knowledge sharing is recognized and rewarded. Communities must develop strategies to engage institutional stakeholders and build support for cultural change if these efforts are to be sustainable.

Some shifts have already begun. Initiatives like the DORA Researcher Development Concordat, which many funders and universities have signed up to, highlight the need to better reward the diverse contributions that researchers make beyond publications alone, as well as shape an environment that enables a more sustainable research culture. Furthermore, the rapid and sustained growth of pre-print servers like bioRxiv and repositories like Addgene (Table 1) have been recognised by the bioscience community as hugely accelerating the pace at which new findings and tools can get into the hands of other researchers^68^, helping build a reputation that benefits both old and new reward systems.

## Towards a connected future

**Fig. 1:**
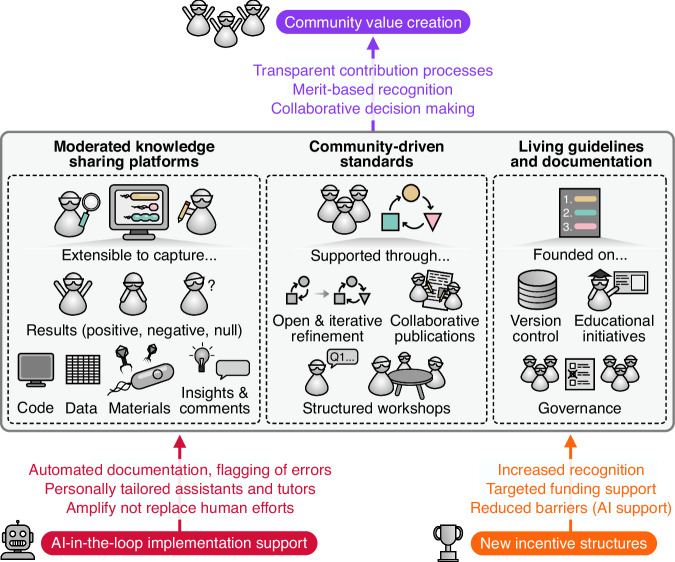
Components for the sustainable preservation of scientific knowledge and skills. We propose a set of three core initiatives (bold) that can be supported by new incentive structures (orange, bottom right) and AI-in-the-loop implementation support (red, bottom left) to enable community value creation (purple, top) and the creation of more sustainable knowledge preservation processes and systems. A selection of some of the key activities are shown.

The challenges outlined in this work point towards a future in which knowledge preservation is not an afterthought but an integral part of the research process itself. Realizing this vision requires building an interconnected ecosystem in which researchers, institutions, and computational systems work together seamlessly (Fig. 1). At its core, this ecosystem would link experimental and computational outputs, null results, tacit expertise, and published findings into a coherent, navigable web of scientific knowledge. Rather than relying on any single platform or technology, such a system would emerge from the coordinated adoption of shared standards, federated storage, and community-driven curation, creating a living record of science that grows richer and more useful over time.

AI will play an increasingly important role in maintaining and navigating this ecosystem, but its value will depend on the quality and structure of the knowledge it draws upon. Well-curated, standards-compliant research outputs will enable AI systems to assist with literature synthesis, experimental design, troubleshooting, and identifying gaps in the scientific record. In turn, AI can reduce the burden on individual researchers by automating metadata generation, flagging broken links or deprecated dependencies, and surfacing relevant prior work, including negative results, that might otherwise remain hidden. This reciprocal relationship, in which better-preserved knowledge enables more capable AI, and more capable AI lowers the cost of preservation, has the potential to create a “flywheel” for continual improvement. However, human judgment must remain central. Researchers will continue to provide the contextual understanding, critical evaluation, ethical guidance, and creative insight that no automated system can replicate, and governance structures must ensure that AI augments rather than supplants the expertise of the research community.

Crucially, the success of this vision depends on people. Technology alone cannot solve what is fundamentally a cultural and organizational challenge. Researchers must be supported, incentivized, and given protected time to contribute to knowledge preservation as a core professional activity. Institutions and funders must recognize these contributions in hiring, promotion, and grant decisions. Scientific societies must continue to foster the networks through which technical knowledge flows, while actively lowering barriers to participation for early-career researchers and those from less well-resourced settings. Cross-disciplinary collaboration will be essential, not only for developing tools and standards that serve diverse communities, but for cultivating the shared understanding needed to tackle problems that no single discipline can address alone.

The path forward will be incremental rather than revolutionary, built on the pragmatic integration of existing tools, the gradual adoption of new platforms, and sustained investment in community engagement. No single initiative will be sufficient; progress will come from many coordinated efforts, each contributing to a more complete and resilient scientific record. By acting now to strengthen the connections between people, data, and machines, the research community can ensure that the knowledge generated today remains accessible, interpretable, and useful for generations to come, supporting a scientific enterprise that is not only more productive but also more open, equitable, and robust.
